# Automatic Detect Incorrect Lifting Posture with the Pose Estimation Model

**DOI:** 10.3390/life15030358

**Published:** 2025-02-24

**Authors:** Gee-Sern Jison Hsu, Jie Syuan Wu, Yin-Kai Dean Huang, Chun-Chieh Chiu, Jiunn-Horng Kang

**Affiliations:** 1 Department of Mechanical Engineering, National Taiwan University of Science and Technology, Taipei 10607, Taiwan; jison@mail.ntust.edu.tw; 2 School of Medicine, College of Medicine, Taipei Medical University, Taipei 11031, Taiwan; b101109092@tmu.edu.tw; 3 Department of Physical Medicine and Rehabilitation, Taipei Medical University Hospital, Taipei 11031, Taiwan; 4 Department of Physical Medicine and Rehabilitation, Wan Fang Hospital, Taipei Medical University, Taipei 11696, Taiwan; 5 Department of Physical Medicine and Rehabilitation, School of Medicine, College of Medicine, Taipei Medical University, Taipei 11031, Taiwan; 6 Graduate Institute of Nanomedicine and Medical Engineering, College of Biomedical Engineering, Taipei Medical University, Taipei 11031, Taiwan

**Keywords:** occupational back injury, lifting posture, camera, markerless system, pose estimation, artificial intelligence

## Abstract

**Background:** Occupational low back pain (LBP) is a pervasive health issue that significantly impacts productivity and contributes to work-related musculoskeletal disorders (WMSDs). Inadequate lifting postures are a primary, modifiable risk factor associated with LBP, making early detection of unsafe practices crucial to mitigating occupational injuries. Our study aims to address these limitations by developing a markerless, smartphone-based camera system integrated with a deep learning model capable of accurately classifying lifting postures. **Material and Method:** We recruited 50 healthy adults who participated in lifting tasks using correct and incorrect postures to build a robust dataset. Participants lifted boxes of varying sizes and weights while their movements were recorded from multiple angles and heights to ensure comprehensive data capture. We used the OpenPose algorithm to detect and extract key body points to calculate relevant biomechanical features. These extracted features served as inputs to a bidirectional long short-term memory (LSTM) model, which classified lifting postures into correct and incorrect categories. **Results**: Our model demonstrated high classification accuracy across all datasets, with accuracy rates of 96.9% for Tr, 95.6% for the testing set, and 94.4% for training. We observed that environmental factors, such as camera angle and height, slightly influenced the model’s accuracy, particularly in scenarios where the subject’s posture partially occluded key body points. Nonetheless, these variations were minor, confirming the robustness of our system across different conditions. **Conclusions:** This study demonstrates the feasibility and effectiveness of a smartphone camera and AI-based system for lifting posture classification. The system’s high accuracy, low setup cost, and ease of deployment make it a promising tool for enhancing workplace ergonomics. This approach highlights the potential of artificial intelligence to improve occupational safety and underscores the relevance of affordable, scalable solutions in the pursuit of healthier workplaces.

## 1. Introduction

Occupational low back pain is a prevalent health problem with a multifactorial etiology that reduces the productivity of manual material handling (MMH) workers and increases the risk of work-related musculoskeletal disorders. According to Hoy et al., the incidence of low back pain ranges from 6.3% to 15.4%, while the recurrence rate ranges from 24% to 80% [1], highlighting its significance in society. Inadequate lifting posture is recognized as a modifiable risk factor for occupational low back pain (LBP) [2]. Early detection of high-risk lifting postures among MMH workers can help minimize the risk and reduce the incidence of occupational injuries.

Direct visual observation, the motion capture system [3,4], the force plate [5], and the measurements from wearable devices such as inertial measurement units (IMUs) [6,7] are common methods to evaluate lifting postures. While visual observation is a simple method, it can be easily affected by subjectivity, and conducting it in a real-world scenario can be time-consuming and impractical. Motion capture systems and IMUs can provide precise and objective measurements in capturing and recording body motion during work. However, applying them in real-world settings is challenging due to the additional time required for setup, inconvenient use, and the relatively high equipment cost.

With advanced deep learning models and computer vision technology, markerless human posture-estimation systems based on a common camera have become a feasible and reliable solution in several fields [8,9]. Lifting is an important issue related to injuries of MMH workers; many researchers have used deep learning models applied to lifting videos or images to investigate lifting posture assessment or estimate lifting load on the lower back [10,11,12,13]. Several different algorithms for pose estimation have been published over the past decade, such as OpenPose [14,15], DeepLabCut [16], DeepPose [17], DeeperCut [18], Alpha Pose [19], and ArtTrack [20]. OpenPose is a well-known open-source library and has been adopted by many researchers and various applications in recent years [21,22,23,24,25]. OpenPose utilizes a unique architecture that combines convolutional neural networks with a part affinity field to accurately identify and track body parts across multiple individuals. It is designed for real-time multi-person 2D pose estimation, enabling the detection of anatomical landmarks of the human body, including hand, facial, and foot key points from images and videos [14].

Integrating original surveillance camera systems with AI can help detect high-risk behaviors and reduce potential occupational hazards and injuries in the workplace. Furthermore, the AI-based model can perform real-time monitoring and recording, enabling the estimation of the cumulative risk in the workplace. Therefore, the present study aims to develop an AI model that uses a smartphone camera to capture lifting motions, followed by automatic analysis and classification of the lifting posture in a simulated scenario. The model can be implemented in the workplace to reduce lower back pain caused by inadequate lifting postures.

## 2. Materials and Methods

### 2.1. Participants

Eligible participants were aged ≥ 20 years old without a history of back disease or without any back injuries in the last year. Participants should be able to cooperate with the instructions to complete the task. Participants were excluded if they had deformities of the body, had undergone previous surgery at the lumbar, sacral, spinal, chest, or abdominal areas, and had osteoporosis, cognitive dysfunction, or mental illness. The study complied with international ethical guidelines. All patients provided written informed consent. The Taipei Medical University Joint Institutional Review Board approved the study (N202201032 & N202307009). Table 1 shows the demographic data of the participants.

### 2.2. Procedures

We recruited 50 healthy volunteers from Taipei Medical University. We asked participants to lift three boxes six times: three times with correct lifting posture and three times with incorrect posture. Their postures were recorded from three directions and two heights of a camera to build a training dataset. We used the videos of 25 participants as training dataset Tr for model construction and the rest as testing dataset Ts for model validation. We collected the external validation dataset Ty, which included 25 lifting videos with the keyword “manual lifting” on YouTube to test the model’s accuracy in various real-world environments.

**Figure 1:**
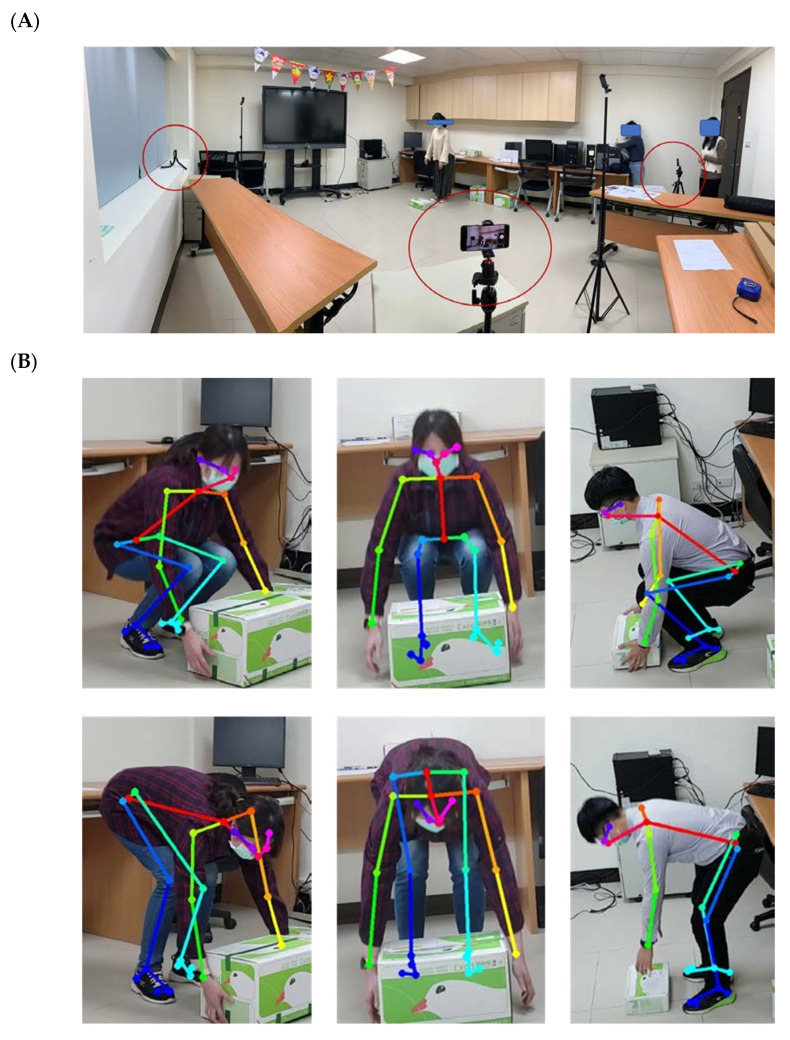
(**A**) The setting of the recording environment of the study. The camera was placed at three different angles and heights to simulate the conditions of a real-world workplace. (**B**) The participants were asked to lift the box with correct lifting posture (above row) and incorrect lifting posture according to The National Institute for Occupational Safety and Health (NIOSH) guidelines.

The lifting guidelines are based on the National Institute for Occupational Safety and Health (NIOSH) lifting recommendations [26]. Correct lifting postures involve keeping the spine as upright as possible while bending the knees to reach the level of the object. On the other hand, incorrect lifting postures include lifting with fully extended knees and a bent torso. An experienced physiatrist did video labeling according to the guidelines. Figure 1 demonstrates the recording environment and examples of correct/incorrect lifting postures.

**Figure 2:**
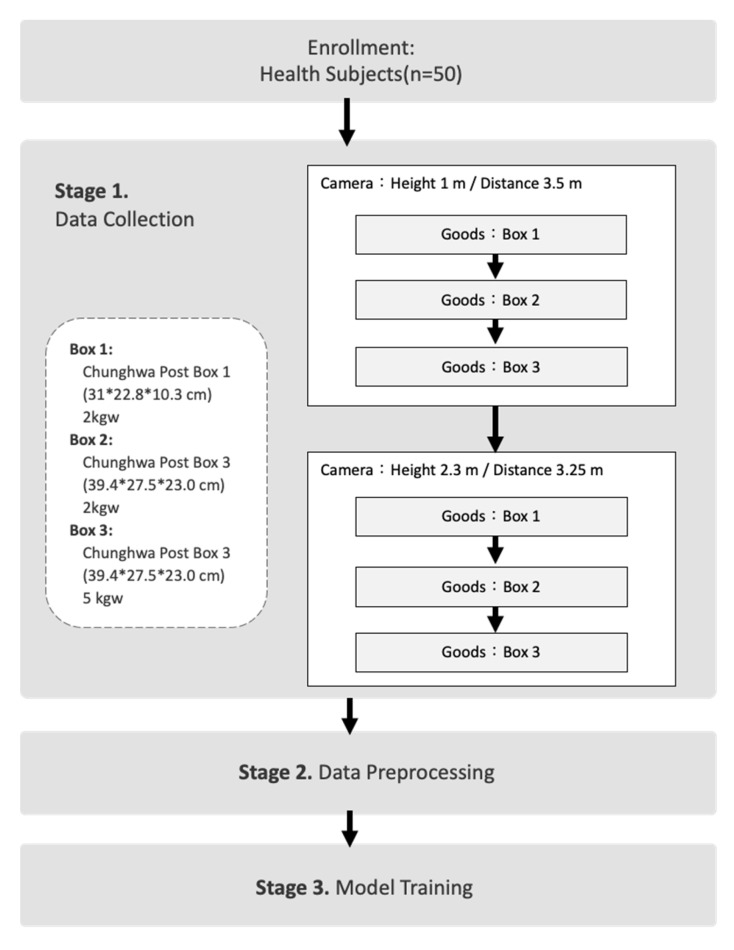
The experimental flow protocol.

We used smartphones and tripods to set up the camera recording system. Video resolution is 1920 × 1080 ppi, with 30 frames per second (fps). We set the camera at three angles: frontal plane (0 degrees), sagittal plane (90 degrees), and oblique plane (45 degrees). The two camera settings were as follows: first, height of 1.0 m from 3.5 m; second, height of 2.3 m from 3.5 m. Three cameras (three directions) with the same height were taking videos simultaneously. Participants lifted three kinds of boxes. Box 1 is 31 × 22.8 × 10.3 cm with a total weight of 2 kg, box 2 is 39.4 × 27.5 × 23.0 cm with a total weight of 2 kg, and box 3 is Chunghwa Post Box 3 with a total weight of 5 kg. The experimental protocol is shown in Figure 2.

We established a video recording system using smartphones mounted on tripods. The videos were captured at a resolution of 1920 × 1080 ppi with 30 frames per second (fps). The raw frames were then directly fed into OpenPose for key point extraction. The recording procedure involved two camera height settings: first at a height of 1.0 m from a distance of 3.5 m, and then at a height of 2.3 m from a distance of 3.25 m. At each height setting, participants were asked to lift three different boxes individually while being recorded from three angles: frontal plane (0 degrees), oblique plane (45 degrees), and sagittal plane (90 degrees). For each box, participants demonstrated both correct and incorrect lifting postures.

The three boxes used in the experiment had the following specifications:

- Box 1: 31 × 22.8 × 10.3 cm, weighing 2 kg
- Box 2: 39.4 × 27.5 × 23.0 cm, weighing 2 kg
- Box 3: Chunghwa Post Box 3, weighing 5 kg

This experimental protocol is illustrated in Figure 2.

### 2.3. Data Preprocessing

We utilized OpenPose v1.3 [14,27] to extract anatomical landmarks from the video frames, which are represented as key points. These key points provided the basis for calculating specific kinematic features, including the angles, angular velocities, and accelerations of the shoulder joint, hip joint, and knee joint.

To capture the posture characteristics during lifting tasks, we incorporated an additional feature based on the difference in y-coordinates between the neck point and the middle hip point. This metric reflects the alignment of the trunk. Specifically, when performing lifting with proper posture, the trunk typically maintains a more upright position, resulting in a larger y-coordinate difference. Conversely, during improper lifting, excessive trunk flexion leads to a smaller y-coordinate difference.

To handle data quality issues, we implemented a preprocessing approach using a time window of 15 frames, which is equivalent to 0.5 s. Within this window, missing key points, instances, where OpenPose failed to detect landmarks or outlier values, and coordinates exceeding two standard deviations from the time window mean, were identified. These erroneous values were substituted with the mean coordinates calculated from the respective time window, thereby minimizing noise and improving data consistency.

### 2.4. Statistical Analysis

Statistical analyses were performed using R version 4.1.0 [28] through RStudio version 2023.12.1 (build 402) interface. To determine appropriate statistical tests, we first assessed the normality of feature distributions using the Kolmogorov-Smirnov test. For features following normal distribution, differences between correct and incorrect postures were analyzed using Two-Sample T-tests. All statistical tests were conducted at a significance level of *p* < 0.05. Features were expressed as means with standard deviations (SD) for normally distributed data and means for non-normally distributed data.

### 2.5. LSTM Classifier

The modeling and training for this study were conducted on a workstation equipped with an NVIDIA RX-4096 GPU. The system operated on an Ubuntu 20.04 LTS Linux environment, and the implementation of the study’s methodologies was carried out using Python 3.1.1. The decision to use an LSTM model was based on its extensive application in medical research, where it has consistently demonstrated robust performance in time-series data analysis. Furthermore, empirical evidence supports its reliability and accuracy across a wide range of scenarios, making it a well-established choice. Additionally, while newer models may offer improved accuracy, their computational requirements are significantly higher. Given our goal of developing a lightweight and efficient model, the LSTM model was ideal for balancing performance and computational efficiency.

We developed three separate LSTM models to analyze lifting postures from different viewing angles: frontal plane (0 degrees), oblique plane (45 degrees), and sagittal plane (90 degrees). Each model follows identical architecture but is trained independently on angle-specific data, enabling comparative analysis of view-dependent performance characteristics.

To optimize the hyperparameters of the LSTM model for sequence classification tasks, we employed a systematic methodology. These included the number of LSTM units per layer, batch size, time step size, and learning rate. The range for each hyperparameter was selected based on preliminary experiments and domain knowledge, with values for LSTM units ranging from 32 to 128 in increments of 32, batch sizes set to 32 and 64, time step sizes of 32 and 64, and learning rates of 0.001 and 0.0001. We utilized grid search, which systematically explored possible combinations of the specified hyperparameters. Each configuration was trained for ten epochs using the training dataset. The performance of the best-performing model was identified based on the lowest observed training loss. Early stopping was employed to terminate training when validation loss did not improve for three consecutive epochs. Additionally, a learning rate scheduler was applied to reduce the learning rate by a factor of 0.5 when the validation loss plateaued. The best-performing model configuration was saved for further analysis and deployment.

Each model comprises a bidirectional LSTM network implemented using TensorFlow 2.11 [29,30]. The network consists of two sequential LSTM layers, each comprising 64 neurons with tanh activation functions. The bidirectional configuration enables the model to process sequential data in both forward and backward directions, capturing temporal dependencies more effectively. The output from the LSTM layers feeds into a dense layer utilizing softmax activation. This layer contains four neurons: two for the classification of correct and incorrect postures, one for non-lifting states, and one for padding sequences. The model processes input sequences in batches of 64 frames, with each sequence containing 64 time steps. Classification output is determined by applying a threshold of 1.5 to the model’s predictions. For optimization, we employ the Adam optimizer with sparse categorical cross entropy as the loss function. The model was trained for 100 epochs, with the dataset split into training and validation sets in an 8:2 ratio. The architecture can be represented mathematically as follows:

Let $e_{j}=\left[e_{1},\;e_{2},\;\ldots ,\;e_{n}\right]$ and $g_{j}=\left[g_{1},\;g_{2},\;\ldots ,\;g_{n}\right]$ denote the estimated and ground-truth lifting postures for video *j*, respectively. For each frame *x*, $e_{x}$ represents the estimated posture, while $g_{x}$ represents the ground-truth label; 1 is for correct, and 2 for incorrect, posture. The classification output $c_{j}=\left[c_{1},\;c_{2},\;\ldots ,\;c_{n}\right]$ contains binary values where *cx* ∈ {1, 2}. Performance metrics are calculated using

(1) $$\mathrm{average}\;\mathrm{Euclidean}\;\mathrm{distance}=\frac{1}{n}\sum_{i=1}^{n}\|e_{j},\;g_{j}\|$$

(2) $$\mathrm{accuracy}=\frac{\theta }{n}$$

where *n* represents the total frame count, ‖.‖ denotes Euclidean distance, and θ indicates the count of correct classifications (zeros in array $\left|c_{j}-g_{j}\right|$).

This multi-angle approach allows us to evaluate the relative importance and effectiveness of each viewing perspective in lifting posture classification. By comparing performance metrics across the three models, we can identify which viewing angles provide the most reliable and accurate assessments of lifting technique.

## 3. Results

### 3.1. Feature Selection and Statistical Analysis

Statistical analysis identified key distinguishing features between correct and incorrect lifting postures (Table 2). After applying Kolmogorov-Smirnov tests for normality distribution, subsequent analyses using two-sample t-tests or Wilcoxon signed-rank tests revealed significant differences (*p* < 0.05) in the angles and coordinates of hip, knee, and shoulder joints. Angular velocity and angular acceleration showed no significant differences between posture types. These findings guided our feature selection, focusing on the three joint angles and the coordinate difference between the neck and hip.

### 3.2. Model Performance

**Figure 3:**
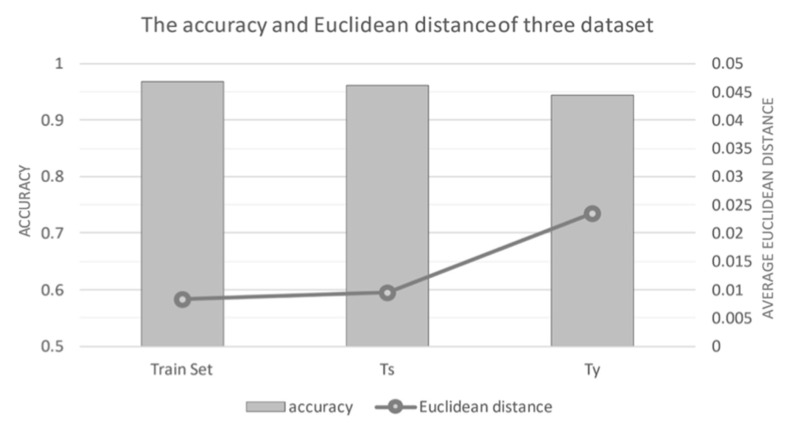
The accuracy and average Euclidean distance of three datasets. Three datasets all have good performance. However, Ty (external validation dataset) has a greater decrease in accuracy and increase in average Euclidean distance, which could be caused by the different video recording conditions.

**Figure 4:**
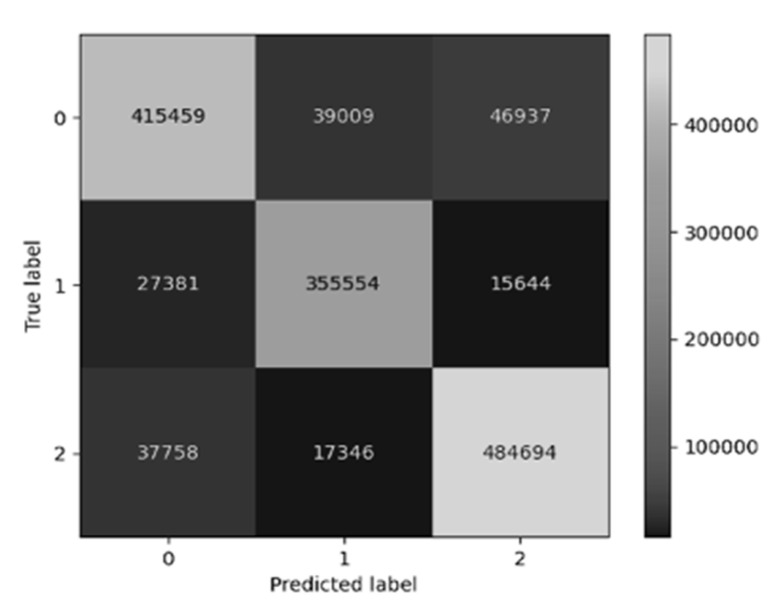
The confusion matrix with heat map of the model. The diagonal grids from upper left to lower right represent the correct predictions of the model. We can find the diagonal grids all have a light color, which means most of the frames are classified into the correct classes (label 0: no life, label 1; lifting with correct posture; label 2: lifting with incorrect posture). The accuracy in classifying correct and incorrect lifting posture is 93.77%, precision is 90.4%, and recall is 95.8%.

Our LSTM model demonstrated exceptional classification accuracy across all datasets (Figure 3). The model achieved 96.9% accuracy in the training set, 95.6% in the validation set (Ts), and 94.4% in the external validation set (Ty). The average Euclidean distances were 0.008, 0.010, and 0.023 for the training, validation, and external validation sets, respectively. The confusion matrix (Figure 4) revealed consistent performance across all datasets, with correct predictions exceeding 94% accuracy along the diagonal axis.

### 3.3. Environmental Impact and Camera Positioning

**Figure 5:**
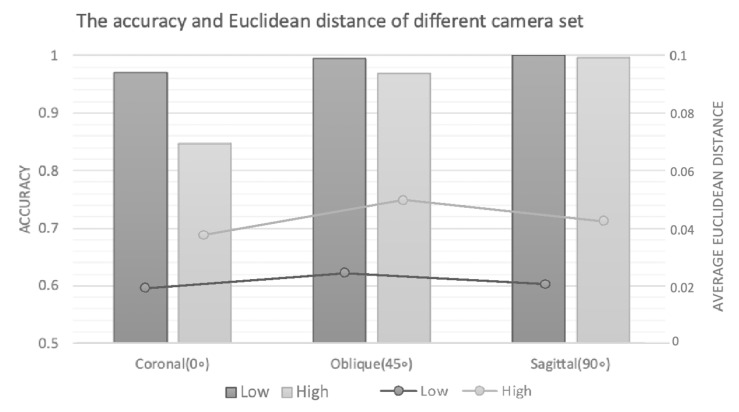
The accuracy and average Euclidean distance of different camera set. L represents the videos shot at a lower camera height (1 m) and H represents the videos shot at a higher camera height (2.3 m). The accuracy in the H group and coronal group is obviously lower, with a higher average Euclidean distance. The joint angle in the coronal group was greatly affected by 3D to 2D projection. Also, since the camera height is greater than the average human height, the H group has a more severe part occlusion problem compared with the L group.

Analysis of environmental factors revealed notable variations in model performance (Figure 4). While maintaining robust accuracy across settings, the external validation dataset (Ty) showed a slight decrease in accuracy and increased average Euclidean distance, reflecting real-world heterogeneity in video recording conditions. Camera positioning analysis (Figure 5) demonstrated that higher camera positions and coronal view recordings resulted in decreased accuracy and increased average Euclidean distance. This performance variation was attributed to two factors: the inherent limitations of 3D-to-2D projection in coronal views and increased part occlusion problems at higher camera positions.

## 4. Discussion

In this study, we have successfully established an LSTM-based method for classifying correct and incorrect lifting postures. The LSTM-based classifier can achieve a high accuracy of 0.969. In line with the study’s primary aim, the findings indicate that the method can accurately determine the lifting posture without needing any markers attached to the participant’s body. In comparison, some scholars have utilized wearable devices with inertial motion units (IMUs) to investigate similar lifting issues in manual laborers. For example, Conforti et al. [6] used eight IMUs attached to the sternum, sacrum, thighs, calves, and feet. Data were collected from 26 healthy participants performing squat lifting and stoop lifting methods. The kinematic data collected during the lifting movements included joint angles of the hip, knee, ankle, and lumbosacral spine, as well as trunk displacement. This study utilized machine learning algorithms to construct a model for classifying the two lifting techniques, achieving an accuracy of 99.4%. This research demonstrates that kinematic data on lifting postures, such as joint angles, can be evaluated and classified using machine learning models. However, the IMU method is inconvenient and needs to be put on IMUs at many specific anatomic landmarks, and IMUs may influence their labor work. Our markerless pose estimation model can also achieve a high accuracy of 0.969.

Markerless pose estimation has the potential to prevent injury and promote health habits. Chen [24] published the use of class monitors to assess students’ sitting posture in class. OpenPose is used to extract the posture features. The Keras deep learning framework is used to construct the convolutional neural network, which is used to recognize correct and incorrect sitting posture of students. The research showed that the accuracy is more than 90% after 100 epochs of training. The author also thinks that it can effectively identify the sitting posture of students in the classroom, which could help young people develop good sitting habits and promote their healthy growth.

Occupational injuries of MMH workers are an important issue and many researchers use pose estimation to investigate this issue. Kim et al. compared OpenPose and kinetic base systems to compute joint angles and RULA/REBA scores and validate them against the reference motion capture system [10]. The research reported that OpenPose is better than the kinetic base system, especially in cases with body occlusions or non-frontal tracking. The findings suggested that OpenPose could be a promising technology for measuring joint angles and conducting semi-automatic ergonomic postural assessments. Zhou et al. proposed a computer vision approach using OpenPose for estimating lifting load contributors to injury risk [13]. Mehrizi et al. used a multi-view-based deep perceptron approach to predict an accurate 3D pose of a lifting task [11]. Mehrizi et al. propose a Deep Neural Network (DNN)-based framework for 3D pose estimation to predict L5/S1 moment and force calculation [12].

The Global Burden of Disease indicates that the prevalence of lower back pain rises to 8.01% [28], and physical workload factors increase the risk of LBP and lumbar radicular pain [31]. Gore et al. analyzed the LifeLink Health Plan Claims Database. They found that patients with chronic low back pain (CLBP) are associated with more significant comorbidity and economic burdens than those without CLBP [32]. An easy-to-use model deployed in the workplace could reduce the burden of medical expenses by early detection of LBP risk factors.

Lifting posture has long been considered a risk factor for lower back pain [33,34,35]. Compared to not being exposed to lifting, lifting loads over 25 kg and lifting at a frequency of over 25 lifts/day will increase the annual incidence of LBP by 4.32% and 3.50% [33]. Occupational lifting is one of the causes of LBP in workers [35]. Gonzalez-Medina et al. report a systematic review to evaluate the effectiveness of the global postural re-education (GPR) program in subjects with persistent chronic low back pain. It provides reliable evidence that GPR may effectively treat low back pain by decreasing pain and improving function [36]. Albaladejo et al. report that adding a short postural hygiene education program in primary care leads to consistent improvements in disability, low back pain, and quality of life [37]. These studies provide strong evidence for posture improvement for treating low back pain, and an inadequate lifting posture detection model could come in handy.

In the present study, we compared asymmetric angle and vertical camera height to understand the effects of camera positions on the model’s performance. The accuracy in the coronal view is lower than in the sagittal and oblique planes, and the lower camera height is more accurate than the higher one; however, there is a low difference in the average Euclidean distance. This represents that the model correctly found the best prediction, but the threshold is not optimized. Due to the various accuracy levels of different camera angles, the threshold should also be modified to match the camera angle. Moreover, the lower accuracy in the high camera position group might have resulted from the partial occlusion of the participant’s body during lifting. The shoulder and head often block the key points of the hip and knee in the coronal plane while the subject stoops down, making the points undetectable in OpenPose and forming a higher classification error.

Our model can be applied over a long period of recording. Calculating accumulated incorrect posture and analyzing workers’ lifting postures could establish a comprehensive database. The incorrect habits of a specific worker can be identified via the daily data collected. Therefore, specific feedback and follow-up to the workers are feasible. This practice potentially benefits all LBP patients’ health and reduces the prevalence of occupational lower back pain. With the database of real-place workers, features such as lifting time, lifting distance, lifting weight, and various lifting methods, including back and head carrying, can be explored in-depth. In the future, several specific features based on key point recognition models can be developed to detect fatigue and estimate the physical conditions of MMH workers. Our model is easy-to-use and has high accuracy. We can deploy it with a simple camera or monitor system as an affordable solution for occupational health.

After building an effective model that is able to automatically detect incorrect posture, this can be used to prevent back injuries among lifting workers. The workplace usually has video monitors. People can build the model into the monitor cameras of the workplace. The system can have the ability to automatically detect workers’ incorrect postures; therefore, the system can complete workers’ ergonomic/posture records and analysis for a specific worker or all the workers. Another application is when the system detects incorrect lifting posture, it can immediately send a feedback message to the specific worker, which can enhance injury prevention.

Several limitations should be addressed. First, this method can only analyze a single person’s lifting posture at a time. Nevertheless, object detection with a bounding box can be applied to overcome this limitation. We can train our region of interest (RoI) detector based on the YOLO model [38,39] to theoretically apply our model in the multipersonal scenario [33,36]. Second, we did not explore the effects of the distance between the box and the body in the lifting task. According to the NIOSH lifting equation [26], the vertical and horizontal distances would affect L5/S1 vertebrae loading. Finally, we only defined correct and incorrect posture in our classification model. Thomas et al. [40] established a DeepConvLSTM-based model with a training set of lifting tasks from 12 lifting zones defined by the ACGIH TLV for lifting [41], and they classified 12 zones as low risk, medium risk, and high risk. The classification recall for low, medium, and high risk is 0.89, 0.84, and 0.96. It is worth noting that increasing recall on high-risk lifting should be a priority rather than considering accuracy in the real-world setting.

## 5. Conclusions

We have established an LSTM-based model with pose estimation on the video recording that automatically detects incorrect lifting postures. With portable instruments and high accuracy, the easy-to-use motion capture system with this model could be applied in various workplaces, reducing the probability of injuries and improving workplace safety.

## Figures and Tables

**Table 1:** Demographic data of participants.

Participant (*n*)	Age (Year)	Height (cm)	Weight (kg)
All (50)	30.4 ± 11.2	166.7 ± 9.2	65.1 ± 14.4
Male (25)	29.8 ± 10.9	173.6 ± 6.5	75.5 ± 12.2
Female (25)	31.0 ± 11.5	159.7 ± 5.6	54.8 ± 7.1

**Table 2:** The features significance analysis. The *p*-value of the angle of hip, knee, and shoulder joints and hip coordinate are all lower than 0.001, reaching significance. According to the significance, we choose these four features as our training features.

Features		Correct			Incorrect			T-Test	Significance
		Mean	SD	K-S Test	Mean	SD	K-S Test		
Angle	hip	131.419	36.074	0.002	124.625	49.071	0.002	0.0008	***
	knee	12.524	4.236	0.002	10.658	2.514	0.002	<0.0001	***
	shoulder	29.552	16.523	0.002	53.524	46.360	0.002	<0.0001	***
Angular Acceleration	hip	9.471	5460.433	0.855	23.932	4188.210	0.814	0.9498	
	knee	11.558	3482.880	0.699	9.303	1568.948	0.634	0.9859	
	shoulder	3.325	3740.386	0.775	1.031	3641.395	0.732	0.9895	
Angular Velocity	hip	0.731	103.279	0.053	0.530	281.535	0.0401	0.9840	
	knee	0.016	71.137	0.002	0.006	31.493	0.004	0.9969	
	shoulder	0.039	80.714	0.033	0.423	270.767	0.114	0.9675	
Coordinate	hip	0.690	0.230	0.002	0.447	0.506	0.002	<0.0001	***

K-S test = Kolmogorov-Smirnov test, T-test = Two-Sample T-test, ** = *p*-value < 0.01, *** = *p*-value < 0.001.

## Data Availability

The data are unavailable due to privacy.
